# Transactions of the Rock-River Medical Society

**Published:** 1849-09

**Authors:** A. M. Catlin

**Affiliations:** Sec’y; Rockford


### ARTICLE VIII.

*Proceedings of the Annual Meeting of the Rock River Medical Society, held at Rockford, May 15th*, 1849.

Pursuant to adjournment, the Rock-River Medical Society met at the Court House in Rockford, at 11 o'clock A. M. The President, Dr. L. Clark, being in the Chair, the Meeting was duly organized by appointing Drs. Van Brunt and Armor Vice-Presidents pro tem., the regular Vice-Presidents being absent.

On motion, The Society adjourned till 1 o'clock P. M.

Pursuant to adjournment, the Society convened at 1 o'clock P. M. The meeting having been called to order, the Records of the last Annual and Semi-Annual Meetings were read.

On motion, Drs. Jeremiah Russel, Charles H. Richings, C. C. Bradley, S. W. Abbott, and John Mitchel, were elected members of this Society.

The President, Dr. L. Clark, delivered an interesting address on Continued Fever, maintaining that we have prevailing, from time to time, in the Valley of the Mississippi, genuine *Typhoid Fever*, having all the characteristics of the "Dothenenteritis" of the French pathologists, the "Abdominal Typhus" of the Germans, and the "Typhoid," so admirably described by Gerhard, Bartlett, and others of this country.

The address was followed by remarks from Prof. Armor, and others, on the peculiar pathological lesions of this form of fever, and the identity and classification of all fevers based on the cause (specified or otherwise) which produced them.

The following cases were then reported:—

1st. Prof. Armor presented a patient laboring under organic disease of the heart, and made some interesting remarks on the general subject of Physical Exploration in diseases of the heart and lungs.

2d. Dr. C. H. Richings reported a case of dropsy from disease of the kidneys.

3d. Dr. D. G. Clark presented an encephaloid cancerous tumor, which he had taken from a female breast, accompanied with interesting remarks.

4th. Dr. L. Clark reported a case of Typhoid Fever, giving its history, &c. On a post mortem examination, the intestines were found perforated.

5th. Dr. A. M. Catlin made some remarks on the subject of Inflammation and Ulceration of the Cervix Uuteri, and on the use of the speculum. He alluded to the fact that uterine diseases were quite prevalent in this region of the country, and not unfrequently very obstinate in character, resisting all the ordinary modes of treatment, being as perplexing and annoying to the physician, as distressing and discouraging to the patient. He maintained that by the use of the speculum, we could arrive at a correct diagnosis *at once*—and from the well known efficacy of the nitrate of silver when applied to inflamed and ulcerated mucous surfaces, we can hope for a speedy and radical cure. Three cases were reported in which cauterization of the cervix with nitrate of silver was employed with the most satisfactory results. This treatment was accompanied by such adjuvants as were indicated by the symptoms present.

6th. Dr. E. N. Clark reported a case of disease of the superior maxillary bone, with suspected disease of the artnum.

The following Resolutions were offered and unanimously adopted :—

By Prof. Armor: *Resolved*, That this society recommend to all its members, and to the physicians throughout the West, the propriety of making as frequent *post-mortem* examinations as possible, as an efficient means of dissipating public prejudice in relation thereto, and advancing the profession in the science of Pathological Anatomy

By Dr. Van Brunt: *Resolved*, That the members of this convention be requested to report severally to the next meeting of the society, all the facts which may come under their observation in reference to *Cholera*—its progress, symptoms, and treatment.

By Dr. Williams: *Resolved*, That Prof. Armor be requested to deliver an address before this society at its next meeting, on *Auscultation* and *Percussion* as a means of diagnosis in diseases of the heart and lungs.

By Dr. Hooker: *Resolved*, That Dr. Lucius Clark be requested to read a paper at the next meeting of the society, on the therapeutical application of the *Chlorate of Potash*.

Drs. J. Mitchell, E. N. Clark, S. W. Abbott, J. S. Lane, and S. Spencer, were appointed Delegates to attend the Wisconsin State Convention, to be held at Janesville on the 15th of June next.

The Chair then appointed Drs. Russel and Martyn to read essays at the next Semi-Annual Meeting.

On motion, it was voted that article IV, of the By-Laws be altered so that six members shall constitute a quorum.

The Society then proceeded to ballot for officers for the ensuing year, which resulted in the following choice:—

President. Dr. A. M, Catlin, of Rockford, Ill.

Vice-Presidents. Drs. C. Van Brunt, of Rockton, Ill.; J. Russel, of Byron, Ill.

Secretary and Treasurer. Dr. C. G. Clark, of Rockford, Ill.

Censors. Drs. A. Clark, of Beloit, Wis.; L. Clark, of Rockford, Ill., and G. Haskell, Rockford, Ill.

On motion: Voted that the next Semi-Annual Meeting be held at Rockton, on the third Tuesday of October next.

On motion: Voted that the next Annual meeting be held at Byron.

On motion: The Society then adjourned till 7 o'clock P. M., at which time they met, and were entertained during the evening, by a lecture from Prof. Armor on the Elements of Disease, as connected with pathological changes of the blood.

On motion: Voted that the thanks of this Society be tendered to Prof. Armor for his able and interesting address.

On motion: The Society then adjourned.

A. M. CATLIN, Sec'y.

Rockford, May 31st, 1849.

